# Yoga in Prevention and Therapy

**DOI:** 10.1155/2017/2160624

**Published:** 2017-01-18

**Authors:** Holger Cramer, Crystal L. Park, Amie Steel, Bangalore N. Gangadhar, Karen Pilkington

**Affiliations:** ^1^Department of Internal and Integrative Medicine, Kliniken Essen-Mitte, Faculty of Medicine, University of Duisburg-Essen, Essen, Germany; ^2^Department of Psychology, University of Connecticut, Storrs, CT, USA; ^3^Endeavour College of Natural Health, Brisbane, QLD, Australia; ^4^Australian Research Centre in Complementary and Integrative Medicine (ARCCIM), Faculty of Health, University of Technology Sydney, Sydney, NSW, Australia; ^5^National Institute of Mental Health and Neurosciences, Bangalore, India; ^6^School of Health Sciences and Social Work, University of Portsmouth, Portsmouth, UK

Yoga is rooted in Indian philosophy and has been a part of traditional Indian spiritual practice for millennia [1]. In recent times, the role of yoga has broadened. Yoga has now also become a popular route to physical and mental well-being and has been adapted for use in complementary and integrative medicine internationally [2]. In the latter setting, yoga most often includes physical postures, breath control, deep relaxation, and meditation/mindfulness techniques. In Western societies, yoga is gaining increased popularity as a preventive and therapeutic practice, making it one of the therapies with the most rapid increase in prevalence [3].

As such, health-care providers are increasingly presented with patients using, or interested in trying, yoga for the management of their health conditions [4]. This increased use of yoga raises issues regarding the efficacy and safety of yoga as a health therapy. Moreover, the potential psychological and physiological mechanisms of action of yoga when used as a preventive and therapeutic modality remain largely unknown. Both efficacy and mechanisms need to be investigated in greater depth in order to inform clinical decision-making and improve research quality regarding one of the most frequently used complementary therapies.

This special issue is dedicated to research that focuses on the clinical application of yoga in preventive medicine and therapy. The peer-reviewed issue solicited original manuscripts in the field of yoga research with a focus on studies that investigated the therapeutic and/or preventive potential of this complementary health-care approach. The accepted manuscripts represent a broad range of research, including clinical trials, qualitative studies, and systematic reviews.

Most papers in this special issue addressed clinical questions and/or the implementation of yoga therapy in different settings. In a randomized controlled trial, Sohl et al. demonstrated beneficial effects of yoga combined with an evidence-based health education program compared to education alone in patients with metabolic syndrome. Positive effects of the combined treatment beyond education alone mainly occurred for physical aspects of quality of life. In a pilot trial, a ten-week yoga therapy intervention induced improvements in joint pain and anxiety in children with cystic fibrosis. This study is important not only because yoga research in pediatric populations is rare but also because it used a one-on-one therapeutic approach, which much more closely resembles the typical yoga therapy setting in clinical practice than the group sessions that are often used in clinical trials. Another study evaluated the feasibility of yoga in a rehabilitation and complex continuing care setting. This study found that yoga adapted for wheelchair users not only was feasible in this setting but also decreased anxiety and pain catastrophizing while increasing self-compassion. Likewise, results of the “Yoga Empowers Seniors Study” demonstrated that yoga could improve physical function in community-dwelling older adults.

Beyond clinical research, this issue features two qualitative studies. Ross et al. interviewed twenty yoga practitioners (three-quarters of them being female) who intentionally or unintentionally had lost weight as a consequence of their yoga practice. They found that, besides physical and psychological changes brought about by yoga practice, changes in eating patterns were perceived as mechanisms of yoga for weight loss. The second qualitative study used focus groups of cancer survivors who had participated in an ongoing community-based yoga program specifically for this patient population. They found that patients valued this disease-specific approach and emphasized the shared understanding and the cancer-specific yoga instructions.

Finally, using a more methodologically oriented approach, a meta-analysis addressed drop-out rates in yoga trials. Based on 168 randomized trials, this analysis provides a guideline for expected drop-out rates when planning randomized controlled trials of yoga interventions.

Several issues remain to be investigated in further research. There are challenges in yoga research such as ideal placebo condition, double-blinding, generic forms of yoga, precise description of the procedure in a given study, and measurements of outcome. Yoga could offer benefits in several domains other than therapy and prevention. This too deserves attention.

We are confident that this special issue, covering a broad spectrum of yoga therapy research, advances the evidence base of this emerging field and usefully informs therapists, health-care providers, and researchers alike.

*Holger Cramer* *Crystal L. Park* *Amie Steel* *Bangalore N. Gangadhar* *Karen Pilkington*
